# Issue Information

**DOI:** 10.1002/iid3.28

**Published:** 2015-03-05

## Aims and Scope

*Immunity, Inflammation and Disease* is a peer-reviewed, open access, interdisciplinary journal providing rapid publication of cutting-edge research across the broad field of immunology. *Immunity, Inflammation and Disease* gives rapid consideration to papers in all are as of clinical and basic research. The journal welcomes original work that enhances the understanding of immunology in areas including cellular and molecular immunology, clinical immunology, allergy, immunochemistry, immunogenetics, cancer immunology, and many others.

Original research articles, methods papers, editorials and commentaries are published. Original research papers must report well-conducted research with conclusions supported by the data presented in the paper. *Immunity, Inflammation and Disease* considers for publication both papers submitted directly to the journal and those referred from a select group of prestigious journals published by Wiley Blackwell. For further information, please refer to our Author Guidelines available at http://onlinelibrary.wiley.com/journal/10.1002/(ISSN)2050-4527/homepage/ForAuthors.html

## Open Access and Copyright

*Immunity, Inflammation and Disease* is a Wiley Open Access journal, one of a series of peer-reviewed titles publishing quality research with speed and efficiency. For further information, please visit the Wiley Open Access website at http://www. wileyopenaccess.com/view/index.html

All articles published by *Immunity, Inflammation and Disease* are fully open access: immediately freely available to read, download and share. Articles are published under the terms of the Creative Commons Attribution License which permits use, distribution and reproduction in any medium, provided the original work is properly cited.

Copyright on any research article in a journal published by *Immunity, Inflammation and Disease* is retained by the author(s). Authors grant Wiley a licence to publish the article and identify itself as the original publisher. Authors also grant any third party the right to use the article freely as long as its integrity is maintained and its original authors, citation details and publisher are identified. Further information about open access license and copyright can be found in this website: http://www. wileyopenaccess.com/details/content/12f25db4c87/Copyright--License.html

## Disclaimer

The Publisher and Editors cannot be held responsible for errors or any consequences arising from the use of information contained in this journal; the views and opinions expressed do not necessarily reflect those of the Publisher and Editors, neither does the publication of advertisements constitute any endorsements by the Publisher and Editors of the products advertised.

Wiley Open Access articles posted to repositories or websites are without warranty from Wiley of any kind, either express or implied, including, but not limited to, warranties of merchantability, fitness for a particular purpose, or non-infringement. To the fullest extent permitted by law Wiley disclaims all liability for any loss or damage arising out of, or in connection with, the use of or inability to use the content.

## Editor-in-Chief

Dr. Marc Veldhoen *Babraham Institute, UK*

Address correspondence to the Editorial Office: Email: IIDeditorial@wiley.com

## Editorial Board

Omid Akbari *University of Southern California, USA*

Mark Coles *University of York, UK*

Anselm Enders *The Australian National University, Australia*

Jochen Huehn *Helmholz Centre for Infection Research, Germany*

Stéphanie Hugues *University of Geneva, Switzerland*

Igor Jurak *Harvard University, USA and University of Rijeka, Croatia*

Ludger Klein *Ludwig Maximilian University of Munich, Germany*

Jun Kunisawa *National Institute of Biomedical Innovation, Japan*

Arian Laurence *Bethesda, MD, USA*

Kathy McCoy *University of Bern, Switzerland*

Osamu Takeuchi *Kyoto University, Japan*

Hai Qi *Tsinghua University, China*

Tim Radstake *University Medical Center Utrecht, Netherlands*

Bruno Silva-Santos *University of Lisbon, Portugal*

Mark Travis *University of Manchester, UK*

David Withers *University of Birmingham, UK*

